# Individualized prediction of survival benefits from perioperative chemoradiotherapy for patients with resectable gastric cancer

**DOI:** 10.1002/cam4.3350

**Published:** 2020-08-18

**Authors:** Keying Che, Fangcen Liu, Nandie Wu, Qin Liu, Ling Yuan, Jia Wei

**Affiliations:** ^1^ The Comprehensive Cancer Centre of Drum Tower Hospital Medical School of Nanjing University & Clinical Cancer Institute of Nanjing University Nanjing China; ^2^ Department of Pathology Nanjing Drum Tower Hospital Clinical College of Nanjing Medical University Nanjing China

**Keywords:** chemotherapy, gastric cancer, prognosis, radiotherapy

## Abstract

**Background:**

The survival benefits of perioperative chemoradiotherapy (PCRT) and perioperative chemotherapy (PCT) for resectable gastric cancer (GC) patients remain unclear. This study aimed to compare the effects of PCRT and PCT in patients with resectable GC and develop a nomogram to evaluate the prognosis and disease risk of patients.

**Methods:**

A total of 6890 patients with stage IB‐IIIC GC from 2010 to 2015 were retrieved from the Surveillance, Epidemiology and End Results (SEER) database. Univariate Cox proportional hazards regression analyses were performed to evaluate the prognostic value of involved variables. A new nomogram was constructed based on development cohort and validated by an external validation cohort. The clinical practicability and accuracy were assessed by concordance index (C‐index), calibration plot, and receiver operating characteristic (ROC) curve.

**Results:**

A better prognosis was obtained for patients with stage III GC treated with PCRT compared with those treated with PCT. Additionally, patients with grade III/IV, diffuse type GC, distal gastric cancer (DGC), tumor size >34 millimeters, or positive lymph nodes were more likely to benefit from PCRT. Multivariate analyses indicated that age, grade, tumor size, T stage, N stage, and comprehensive treatment were independent covariates. Excellent agreement of calibration plots and good discrimination power were obtained using the nomogram. The nomogram achieved a better net benefit than the 8th edition AJCC TNM staging. An online version was built based on the nomogram for convenient clinical use.

**Conclusion:**

The application of perioperative chemoradiotherapy should be determined according to the clinicopathological features of patients. Our nomogram provided a reliable tool for screening patients who were right for PCRT and evaluating individual survival benefits.

## Graphical abstract

The large population‐based study showed that the application of perioperative chemoradiotherapy should be determined according to the clinicopathological features of patients. We further constructed and validated a reliable survival‐predicting nomogram model, with its user‐friendly online web server, to accurately predict individualized survival probability.

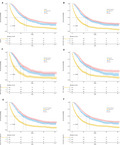

## 1 INTRODUCTION

Gastric carcinoma (GC) is the fifth most common cancer and is the third leading cause of global cancer‐related death. In western countries, the incidence of GC is gradually increasing. Approximately 25 000 new cases are diagnosed each year in the United States.^1^, ^2^ In addition to epidemiologic variations, GC also exhibits heterogeneity in histopathology, molecular biology, and survival prognosis. GC can be categorized by different histological classification systems. The Lauren classification and the World Health Organization (WHO) classification are the most common classification systems. Histological subtypes are known to differ in disease progression and clinical outcome.^3^ Additionally, according to the location of the primary tumor, GC is classified as proximal gastric cancer (PGC) or distal gastric cancer (DGC), which differ in their pathogenesis mechanisms.^4^ Because of the heterogeneity of GC, it is crucial to develop optimal individualized management for patients.

Surgical resection remains the mainstay of treatment for locally advanced GC. However, satisfactory results cannot be achieved solely by surgery. Neoadjuvant and adjuvant therapies are recommended to improve the survival of patients. The benefit of perioperative chemotherapy (PCT) was established by the MAGIC trial and subsequent randomized controlled trials.^5^, ^6^, ^7^, ^8^ On the basis of the obtained results, PCT was shown to be effective for patients, and it became a standard treatment for GC. In consideration of the high local recurrence rate in GC, a combination of radiotherapy and chemotherapy has been proposed and was compared with chemotherapy in several clinical trials. Among these studies, the well‐known INT‐0116 trial and Adjuvant Chemoradiotherapy in Stomach Tumors (ARTIST) trial evaluated the role of postoperative chemoradiation strategy in individuals with resectable GC.^9^, ^10^, ^11^, ^12^ Additionally, the results of the PreOperative therapy in Esophagogastric adenocarcinoma Trial (POET) indicated that the inclusion of radiotherapy in preoperative treatment conferred certain benefits.^13^ However, to our knowledge, no phase III trial has been published in a peer‐reviewed journal comparing preoperative chemotherapy with preoperative chemoradiotherapy (CRT) in patients with resectable GC. In addition, several influential phase III clinical trials, which compared the overall survival (OS) between the postoperative chemotherapy group and the postoperative CRT group, were mainly based on East Asian populations rather than North Americans.^11^, ^12^, ^14^ Until now, it is still unclear whether radiotherapy should be administered with PCT. Therefore, screening to determine which patients are suitable for perioperative chemoradiotherapy (PCRT) is of great significance to improve the survival rate. Because of the very different histopathology, pathogenesis mechanism, and clinical manifestation of GC, searching for clinicopathological features other than tumor‐node‐metastasis (TNM) staging that can also influence patient prognosis is necessary.

The potential prognosis and appropriate treatment strategy are different in populations with different clinicopathologic characteristics. In order to screen patients to determine which ones are suitable for receiving PCRT, we must identify homogeneous high‐risk patient groups. As an available prediction tool, a nomogram can evaluate the prognosis and disease risk of patients. It is a graphical decision‐making tool that can incorporate several variables to predict survival rate and screen high‐, medium‐, and low‐risk groups using statistical methods.^15^ The nomogram has been one of the most widely used clinical prognostic models for malignant tumors.^16^, ^17^

Therefore, this study aimed to evaluate the prognostic value of clinicopathological factors and screening features related to PCRT in patients with GC. We developed a valuable nomogram to predict 1‐, 3‐, and 5‐year survival probability based on the data from the Surveillance, Epidemiology and End Results (SEER) database.

## 2 MATERIAL AND METHODS

### 2.1 Patient screening

**FIGURE 1:**
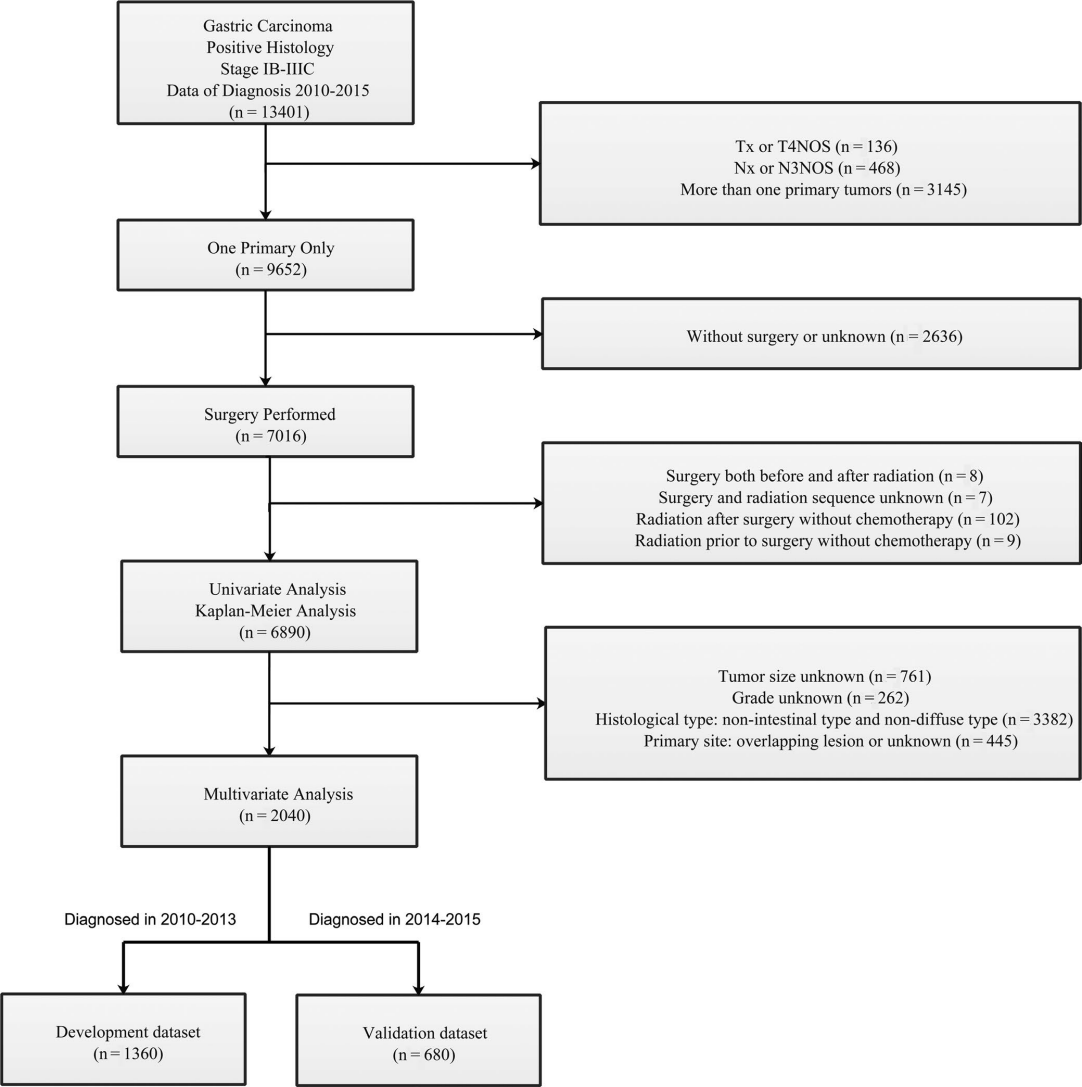
Flowchart of patient selection for this study

Population‐based data of patients with GC were retrieved from 18 registries of the Surveillance Epidemiology and End Results (SEER) Program using SEER*Stat (version 8.3.6). We identified 13,401 patients diagnosed from 2010 to 2015 with stage IB‐IIIC GC (site recode 8.6.2) which were confirmed by histology. GCs were coded by histologic subtype using the International Classification of Diseases for Oncology, 3rd Edition (ICD‐O‐3) (Table S1). Among these cases, patients who met the following criteria were excluded: (a) Tx or T4NOS, (b) Nx or N3NOS, (c) more than one primary tumors, (d) without surgery or unknown, (e) surgery both before and after radiation, (f) surgery and radiation sequence unknown, (g) radiation after surgery without chemotherapy, (h) radiation prior to surgery without chemotherapy. A total of 6890 cases were included for univariate analyses and Kaplan‐Meier analyses. Furthermore, after excluding the following ineligible cases: (a) tumor size unknown, (b) grade unknown, (c) histological type: nonintestinal type and nondiffuse type, (d) primary site: overlapping lesion or unknown, 2040 observations were included for multivariate analyses. 1360 patients (approximately two‐thirds of the dataset) who were diagnosed between 2010 and 2013 were used as the development cohort to construct predictive models, whereas the remaining 680 patients (who were diagnosed between 2014 and 2015) were used as the validation cohort. Figure 1 is the flowchart of patient selection.

### 2.2 Study variables

Following clinical variables from the cohort were extracted: gender, age at diagnosis, marital status, grade, histological type, position of primary tumor, tumor size, T stage, N stage, radiotherapy, chemotherapy, and comprehensive treatment. The continuous variable, “tumor size” was transformed into categorical variable based on the ROC curve and Youden index using MedCalc (Figure S1).^18^, ^19^ The AJCC 7th edition staging in the dataset was transformed into corresponding the 8th edition staging to form the latest data. The primary outcomes of the study were OS and gastric cancer‐specific survival (GCSS). OS was defined as interval between the date of diagnosis and the date of death from any cause or last contact. Time of GCSS was counted from date of diagnosis to date of death due to gastric cancer.

### 2.3 Statistical analyses

The univariate and multivariate analyses were performed using Cox backward stepwise regression model to calculate the hazard ratio (HR) and 95% confidence interval (95% CI) of involved variables. Variables were incorporated into multivariate analyses if they reached a *P* value less than .05 in univariate analyses. The Kaplan‐Meier analyses were used to calculate survival time and survival probability. The survival differences among groups were assessed by log‐rank test. Significance was considered as *P* value less than .05 in a two‐tailed test. Above analyses were performed using SPSS version 20.0 (IBM, SPSS Statistics) and R version 3.6.2 (http://www.r‐project.org/).

A nomogram was devised based on the independent prognostic variables according to the above multivariate regression model. Discrimination and calibration were used to assess accuracy of the nomogram.^15^ Discrimination is defined as the ability of a model to correctly distinguish nonevents and events, and is quantified by the Harrell's concordance index (C‐index). Calibration measures the discrepancy between the predicted probabilities and the actual survival and is presented by graphic calibration curves.^20^ Bootstrap analyses with 1000 resamples was used to evaluate the accuracy of the model.^21^ Furthermore, the area under receiver operating characteristic (ROC) curve (AUC) was applied to evaluate the accuracy of 1‐, 3‐ and 5‐year survival predictions. The decision curve analyses (DCA), the net reclassification improvement (NRI), the integrated discrimination improvement (IDI) and time‐dependent ROC curve were used to assess net benefits and reliability of the new model.^22^, ^23^, ^24^, ^25^ Above all analyses were performed using R version 3.6.2 via RStudio software (version 1.2.5033). The “rms,” “survival,” “shiny,” “foreign,” “nricens,” and “time‐ROC” packages were used. This study followed the TRIPOD statement.^26^

### 2.4 Ethical declaration

The study used de‐identified data and adhered to the World Medical Association's Declaration of Helsinki for Ethical Human Research.

## 3 RESULTS

### 3.1 Characteristics of patients

A cohort of 6890 patients with stage IB to IIIC GC diagnosed from 2010 to 2015 was analyzed by univariate Cox regression. Of the 6890 observations, 2040 cases were incorporated into multivariate Cox regression analyses. All cases were confirmed by pathology. The baseline characteristics of the patients and univariate Cox regression analyses are summarized in Table 1. The median age of included patients was 65 years. Of note, patients receiving perioperative chemotherapy (PCT) and perioperative chemoradiotherapy (PCRT) all exhibited significant survival benefits compared with surgery alone. Thus, the characteristics of patients who were suitable for PCRT required further study. Of the 6890 observations, 2040 cases who met the criteria were incorporated into multivariate Cox regression analyses. With the results from the multivariate analyses model, we produced a nomogram to predict the survival probability.

**TABLE 1:** Patients’ characteristics and univariate analyses of overall survival and gastric cancer‐specific survival

Variables	Count (%)	Overall survival (OS)		Gastric cancer‐specific survival (GCSS)	
		HR (95% CI)	*P* value	HR (95% CI)	*P* value
Gender	6890				
Male	4414 (64.1)	1	—	1	—
Female	2476 (35.9)	0.989 (0.922‐1.061)	.763	1.313 (1.205‐1.431)	<.001
Age at diagnosis	6890				
<65	3275 (47.5)	1	—	1	—
≥65	3615 (52.5)	1.501 (1.401‐1.607)	<.001	1.448 (1.329‐1.579)	<.001
Marital status	6517				
Married	5487 (84.2)	1	—	1	—
Unmarried	1030 (15.8)	1.021 (0.929‐1.123)	.667	1.012 (0.897‐1.142)	.849
Grade	6540				
Grade I/II	2005 (30.7)	1	—	1	—
Grade III/IV	4535 (69.3)	1.545 (1.426‐1.674)	<.001	2.092 (1.874‐2.334)	<.001
Histological type	2858				
Intestinal type	1029 (36.0)	1	—	1	—
Diffuse type	1829 (64.0)	1.382 (1.239‐1.542)	<.001	1.485 (1.307‐1.688)	<.001
Location of primary tumor	5836				
DGC	3620 (62.0)	1	—	1	—
PGC	2216 (38.0)	0.892 (0.826‐0.963)	.004	0.323 (0.285‐0.367)	<.001
Tumor size	6129				
≤34	2163 (35.3)	1	—	1	—
>34	3966 (64.7)	1.763 (1.625‐1.912)	<.001	2.393 (2.142‐2.674)	<.001
8th T stage	6890				
T1	538 (7.8)	1	—	1	—
T2	1331 (19.3)	0.987 (0.824‐1.183)	.890	1.145 (0.861‐1.522)	.353
T3	3232 (46.9)	1.881 (1.602‐2.208)	<.001	2.607 (2.023‐3.361)	<.001
T4	1789 (26.0)	3.464 (2.944‐4.077)	<.001	7.170 (5.567‐9.233)	<.001
8th N stage	6890				
N0	2268 (32.9)	1	—	1	—
N1	1988 (28.9)	1.431 (1.301‐1.574)	<.001	1.631 (1.425‐1.868)	<.001
N2	1375 (19.9)	1.996 (1.807‐2.205)	<.001	2.707 (2.365‐3.100)	<.001
N3	1259 (18.3)	3.297 (2.996‐3.628)	<.001	5.813 (5.130‐6.587)	<.001
Radiotherapy	6890				
Surgery alone	4028 (58.5)	1	—	1	—
Radiation after surgery	1675 (24.3)	0.804 (0.741‐0.872)	<.001	0.888 (0.808‐0.976)	.014
Radiation prior to surgery	1143 (16.6)	0.866 (0.788‐0.952)	.003	0.233 (0.191‐0.285)	<.001
Radiation before and after surgery	44 (0.6)	1.096 (0.733‐1.639)	.655	0.320 (0.133‐0.770)	.011
Chemotherapy	6890				
No/unknown	2336 (33.9)	1	—	1	—
Yes	4554 (66.1)	0.708 (0.661‐0.759)	<.001	0.664 (0.608‐0.725)	<.001
Comprehensive treatment	6890				
Surgery alone	2336 (33.9)	1	—	1	—
PCT	1692 (24.6)	0.696 (0.636‐0.762)	<.001	0.820 (0.737‐0.912)	<.001
PCRT	2862 (41.5)	0.715 (0.662‐0.772)	<.001	0.576 (0.521‐0.636)	<.001

Abbreviations: CI, confidence interval; DGC, distal gastric cancer; GCSS, gastric cancer‐specific survival; HR, hazard ratio; N, node; OS, overall survival; PCRT, perioperative chemoradiotherapy; PCT, perioperative chemotherapy; PGC, proximal gastric cancer; SEER, Surveillance, Epidemiology, and End Results; T, tumor.

### 3.2 Subgroup analyses

**FIGURE 2:**
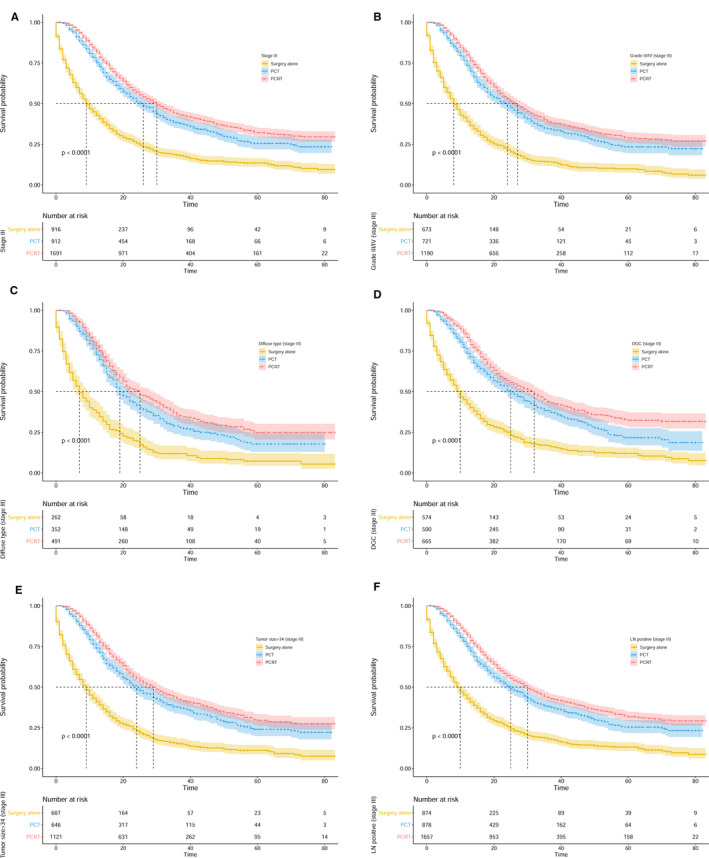
Kaplan‐Meier survival curves for patients with resectable gastric cancer in different subgroups, which are stratified by treatment strategies. The dotted lines indicate median survival time of patients. (A) OS for patients with stage III GC. (B) OS for patients with stage III GC in the grade III/IV subgroup. (C) OS for patients with stage III GC in the diffuse type subgroup. (D) OS for patients with stage III GC in the DGC subgroup. (E) OS for patients with stage III GC in the tumor size >34 mm subgroup. (F) OS for patients with stage III GC in the lymph node‐positive subgroup

**FIGURE 3:**
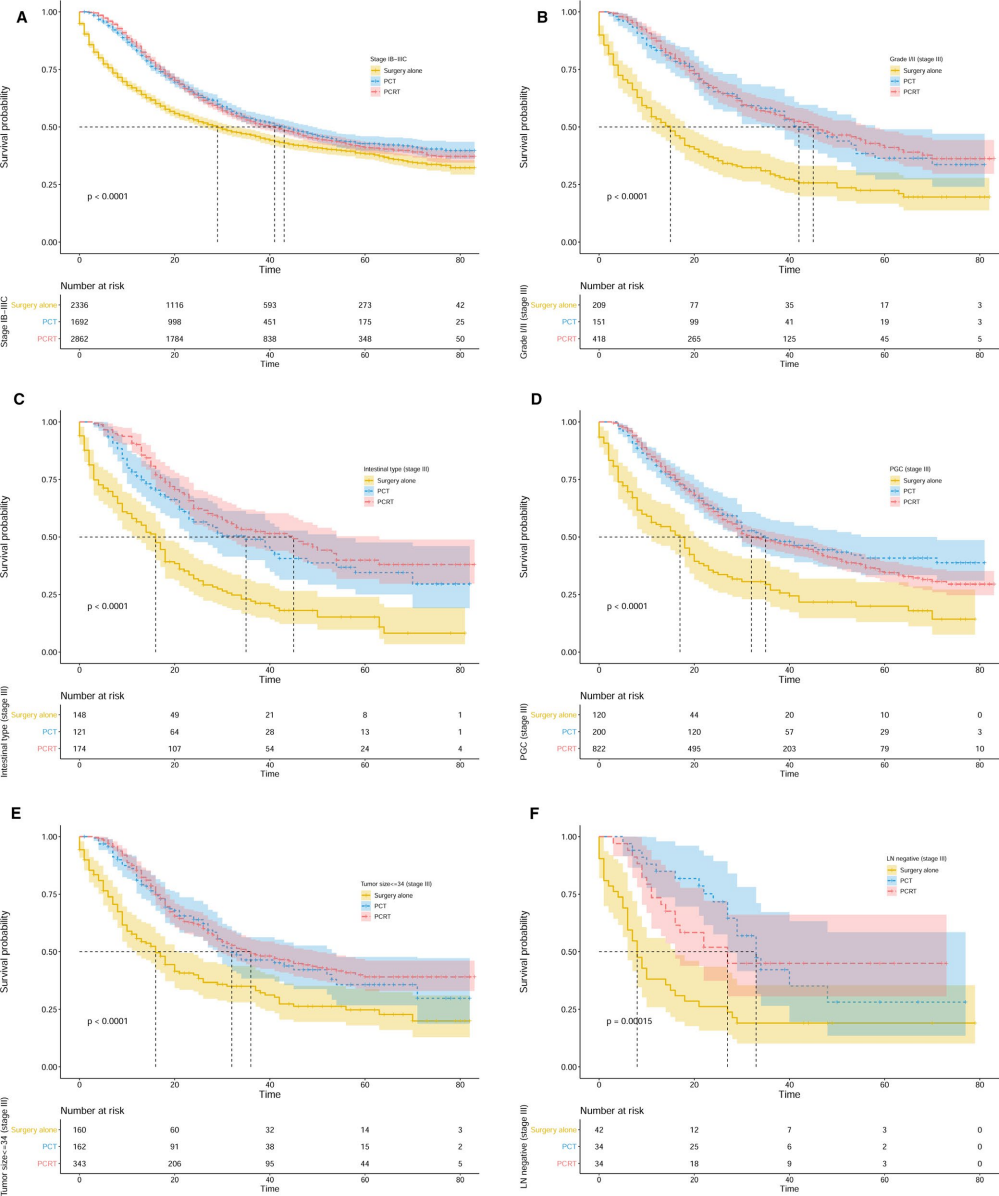
Kaplan‐Meier survival curves for patients with resectable gastric cancer in different subgroups, which are stratified by treatment strategies. The dotted lines indicate median survival time of patients. (A) OS for patients with stage I B‐III C GC. (B) OS for patients with stage III GC in the grade I/II subgroup. (C) OS for patients with stage III GC in the intestinal type subgroup. (D) OS for patients with stage III GC in the PGC subgroup. (E) OS for patients with stage III GC in the tumor size ≤34 mm subgroup. (F) OS for patients with stage III GC in the lymph node‐negative subgroup

As we explained earlier, patients receiving PCT or PCRT exhibited increased survival as compared to patients that received surgery alone. To identify the factors related to survival, subgroup analyses stratified by treatment strategies were performed. We merged preoperative chemoradiotherapy and postoperative chemoradiotherapy into PCRT because of their similar outcomes. The results indicated obvious heterogeneity in the role of PCRT on survival across the subgroups. Compared with PCT, PCRT had no significant impact on OS for patients with stage IB‐IIIC GC (43 months vs 41 months, *P* = .685) (Figure 3A). Nevertheless, the OS and GCSS of stage III patients with PCRT were significantly longer than those with PCT (OS: 26 months vs 30 months, *P* < .001; GCSS: 32 months vs —, *P* < .001) (Figure 2A; Figure S2A). Additionally, grade, histology type, position of the primary tumor, tumor size, and lymph node status were all included to evaluate their prognostic impact. For patients with stage III GC, those with grade III/IV, diffuse type, DGC, tumor size >34 millimeter (mm), or lymph node‐positive disease were more likely to benefit from PCRT. The survival analyses showed that patients with grade III/IV GC receiving PCRT exhibited much better survival than those receiving PCT (OS: 24 months vs 27 months, *P* = .004; GCSS: 29 months vs 53 months, *P* < .001) (Figure 2B; Figure S2B). As shown in Figure 2C and Figure S2C, PCRT significantly improved survival for patients with diffuse type GC (OS: 19 months vs 25 months, *P* = .008; GCSS: 22 months vs 32 months, *P* = .001). PCRT exerted a more optimal impact on survival in patients with DGC (OS: 25 months vs 32 months, *P* = .001; GCSS: 29 months vs 36 months, *P* = .002), with tumor size >34 mm (OS: 24 months vs. 29 months, *P* = .002; GCSS: 30 months vs 58 months, *P* < .001) or patients with positive lymph nodes (OS: 25 months vs 30 months, *P* < .001; GCSS: 32 months vs —, *P* < .001) (Figure 2D‐F; Figure S2D‐F). In addition, compared with PCT, PCRT did not significantly influence OS in the patients with grade I/II GC (42 months vs 45 months, *P* = .707), intestinal type GC (35 months vs 45 months, *P* = .157), PGC (35 months vs 32 months, *P* = .420), tumor size ≤34 mm (32 months vs 36 months, *P* = .601), or negative lymph nodes (33 months vs 27 months, *P* = .748) (Figure 3B‐F).

### 3.3 Risk covariates associated with survival in cohorts

Univariate and multivariate Cox proportional hazard models were performed to assess the value of clinical variables on survival. Initially, using univariate analyses, gender, age at diagnosis, grade, histological type, location of primary tumor, tumor size, T stage, N stage, radiotherapy, chemotherapy, and comprehensive treatment were found to be significantly associated with OS and GCSS (Table 1). Remarkably, PCT (OS: HR 0.696, GCSS: HR 0.820) and PCRT (OS: HR 0.715, GCSS: HR 0.576) were associated with increased survival compared with surgery alone.

Furthermore, multivariate analyses were used to identify independent prognostic factors. Table 2 shows that age at diagnosis, grade, tumor size, T stage, N stage, and comprehensive treatment were all independent prognostic variables in GC patients.

**TABLE 2:** Multivariate analyses of overall survival and gastric cancer‐specific survival

Variables	Overall survival (OS)		Gastric cancer‐specific survival (GCSS)	
	HR (95% CI)	*P* value	HR (95% CI)	*P* value
Gender				
Male	1	—	1	—
Female	0.882 (0.776‐1.003)	.055	0.932 (0.804‐1.080)	.346
Age at diagnosis				
<65	1	—	1	—
≥65	1.442 (1.258‐1.653)	<.001	1.408 (1.202‐1.649)	<.001
Grade				
Grade I/II	1	—	1	—
Grade III/IV	1.237 (1.021‐1.500)	.030	1.441 (1.143‐1.816)	.002
Histological type				
Intestinal type	1	—	1	—
Diffuse type	1.149 (0.975‐1.353)	.097	1.091 (0.903‐1.317)	.367
Location of primary tumor				
DGC	1	—	1	—
PGC	1.188 (0.996‐1.417)	.056	0.807 (0.635‐1.025)	.078
Tumor size				
≤34	1	—	1	—
>34	1.283 (1.101‐1.495)	.001	1.330 (1.108‐1.596)	.002
8th T stage				
T1	1	—	1	—
T2	1.390 (0.965‐2.002)	.077	1.569 (0.948‐2.595)	.079
T3	2.342 (1.688‐3.247)	<.001	3.021 (1.923‐4.747)	<.001
T4	3.539 (2.536‐4.940)	<.001	5.041 (3.199‐7.945)	<.001
8th N stage				
N0	1	—	1	—
N1	1.632 (1.341‐1.986)	<.001	1.805 (1.413‐2.305)	<.001
N2	1.879 (1.540‐2.293)	<.001	2.282 (1.792‐2.907)	<.001
N3	2.499 (2.057‐3.037)	<.001	3.009 (2.380‐3.803)	<.001
Comprehensive treatment				
Surgery alone	1	—	1	—
PCT	0.500 (0.422‐0.593)	<.001	0.579 (0.478‐0.701)	<.001
PCRT	0.437 (0.375‐0.510)	<.001	0.437 (0.365‐0.524)	<.001

Abbreviations: CI, confidence interval; DGC, distal gastric cancer; GCSS, gastric cancer‐specific survival; HR, hazard ratio; N, node; NE, not evaluated; OS, overall survival; PCRT, perioperative chemoradiotherapy; PCT, perioperative chemotherapy; PGC, proximal gastric cancer; T, tumor.

### 3.4 Development and validation of the nomogram

**FIGURE 4:**
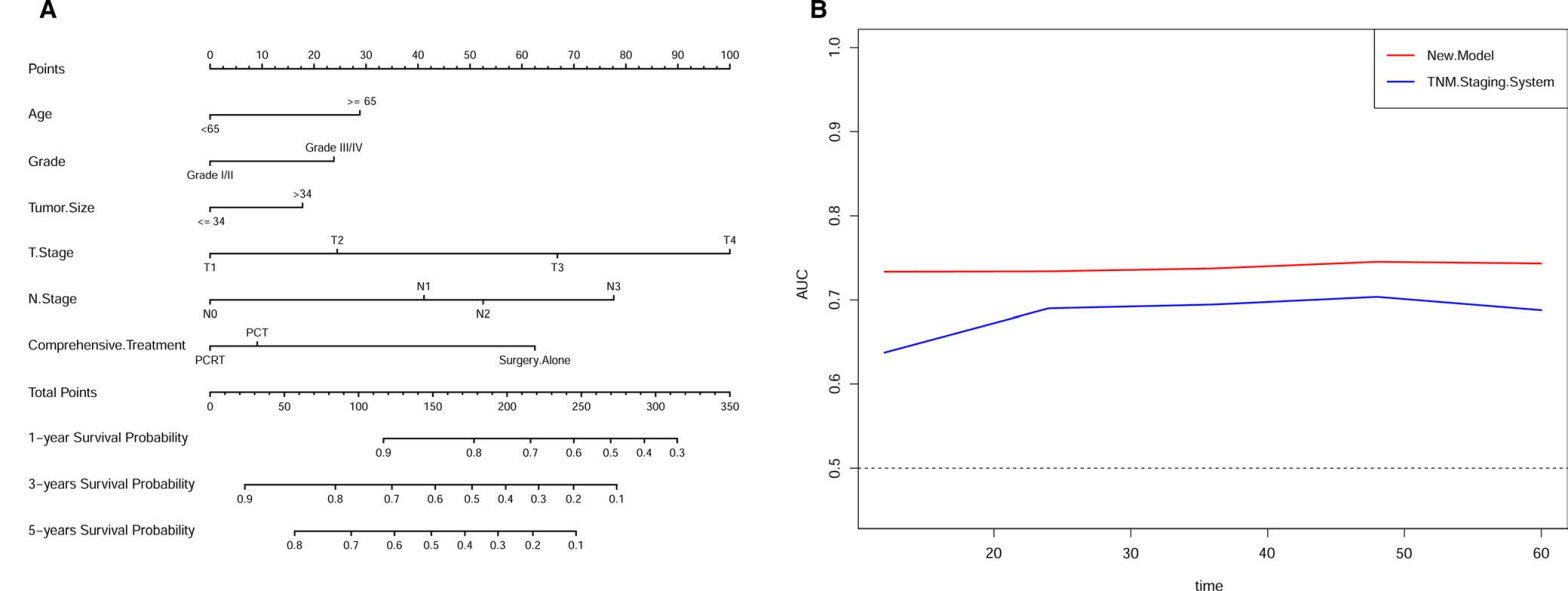
The prognostic nomogram and time‐dependent ROC curve for GC patients. (A) Prognostic nomogram predicting 1‐, 3‐, and 5‐year survival probability for patients with resectable GC using six clinical characteristics. For each predictor, the points assigned on the 0‐100 scale are read at the top, and then, these points are added. The number on the “Total Points” scale is located, and then, the corresponding predictions for 1‐, 3‐, and 5‐year survival probability are read. (B) The time‐dependent ROC curve for GC patients predicted by the new nomogram model (red line) and the 8th TNM staging system (blue line)

The selected variables from the multivariate Cox analyses were used to establish a nomogram to predict the 1‐, 3‐, and 5‐year survival probability (Figure 4A). The six variables were scored by the Points scale ranging from 1 to 100. The nomogram illustrated that the greatest contribution to prognosis was from T stage, followed by N stage, comprehensive treatment, age at diagnosis, grade, and tumor size. Each category of these variables is assigned a score on the Points scale. Total points are calculated by adding all the points from every variable, and the sum is located on the Total Points scale. A line drawn straight down to the 1‐, 3‐, and 5‐year Survival Probability scale reveals the estimated survival probability at each time point.

**FIGURE 5:**
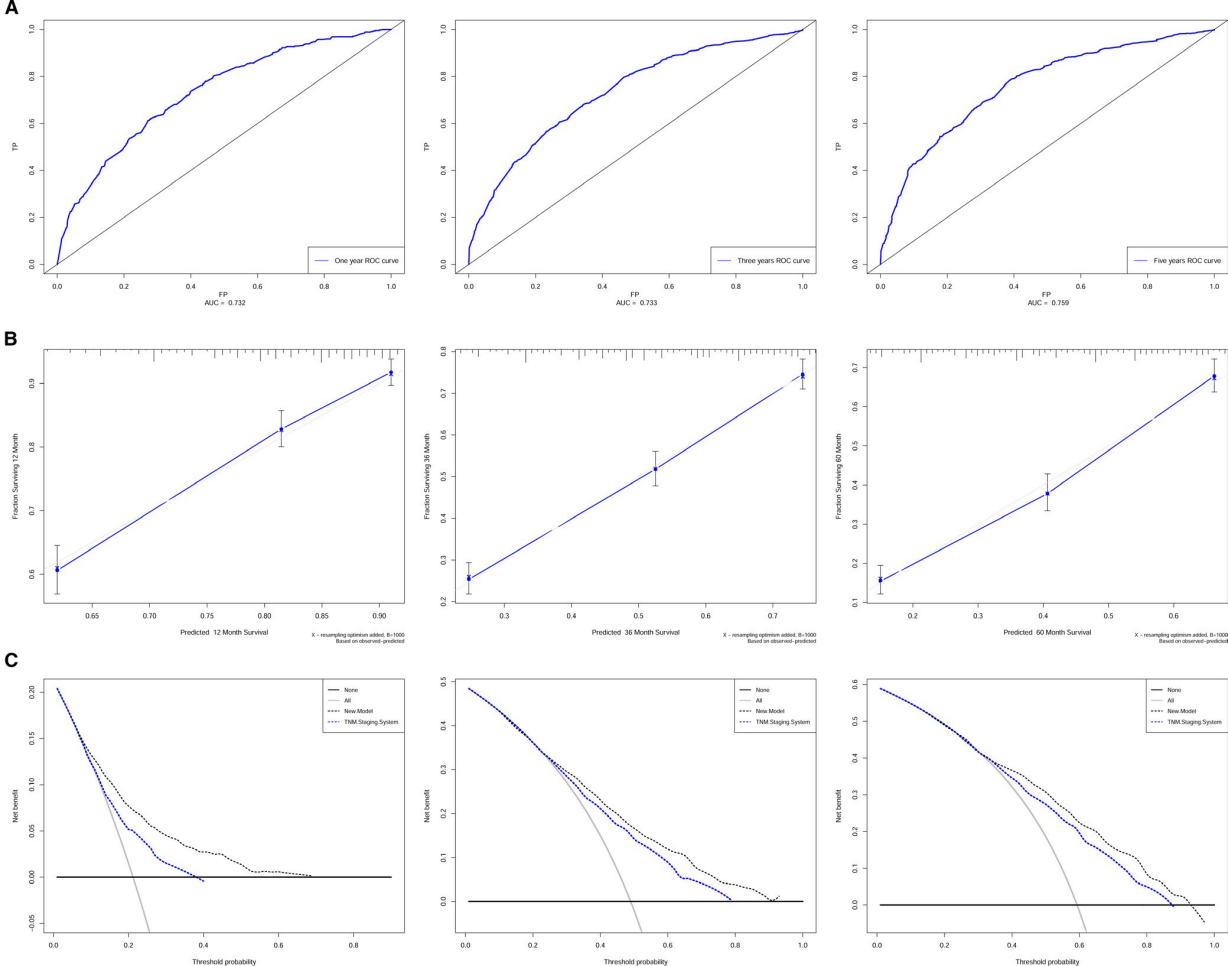
Development dataset of the nomogram for GC patients. (A) ROC curves for 1‐, 3‐, and 5‐year OS in the development cohort. (B) Calibration plots for 1‐, 3‐, and 5‐year OS in the development cohort. (C) DCA curves for 1‐, 3‐, and 5‐year OS in the development cohort

The discrimination and calibration of the nomogram were evaluated using the C‐index and calibration plot. The C‐index of the development cohort was 0.702 (95% CI: 0.693‐0.710, *P* = .009). In the validation cohort, the C‐index was 0.712 (95% CI: 0.694‐0.730, *P* = .018). The C‐indexes for the nomogram were significantly higher than those for the model based on the 8th edition AJCC TNM staging system in both the development (0.702 vs 0.648) and validation (0.712 vs 0.671) cohort (Table S2). Furthermore, the AUC model was built to evaluate the predictive ability of the nomogram. For the development set, the AUCs predicting the 1‐, 3‐, and 5‐year OS rates were 0.732, 0.733, and 0.759, respectively, and the AUCs of the validation set were 0.731, 0.740, and 0.753 for 1‐, 3‐ and 5‐year survival, respectively (Figure 5A; Figure S3A). In addition, calibration plots presented high consistency between nomogram predictions and actual observations (Figure 5B; Figure S3B).

### 3.5 Comparison of the nomogram and the TNM staging system

**FIGURE 6:**
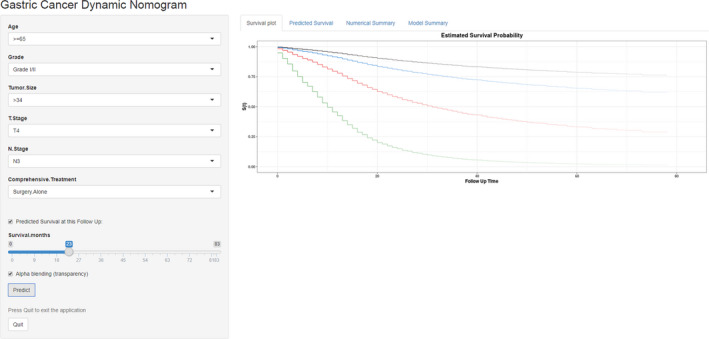
Online web server page of our nomogram

The benefit and reliability of the new model were assessed by comparing it to the 8th edition TNM staging system using DCA, NRI, IDI, and time‐dependent ROC curve. Compared with the TNM staging system, the DCA graphically demonstrated that the new model had more optimal net benefits in predicting the 1‐, 3‐, and 5‐year survival of patients (Figure 5C; Figure S3C). Additionally, in the development cohort, the NRI for the 1‐, 3‐, and 5‐year survival were 0.459, 0.333, and 0.334, and in the validation cohort, the NRI were 0.579, 0.476, and 0.553, respectively. Similarly, analyses showed that the IDI for the 1‐, 3‐, and 5‐year survival were 0.027, 0.032, and 0.029 in the development cohort (all *P* < .001), and 0.046, 0.051, and 0.048 (all *P* < .001) in the validation cohort, respectively. The time‐dependent ROC curve showed that the nomogram had a stronger role for accurately predicting prognosis compared to the TNM staging system (Figure 4B). An online version of the nomogram is available at: https://clinicalprediction.shinyapps.io/Gastric‐Carcinoma/ and can be used to optimize the calculation process in clinical applications (Figure 6). These results indicate that the nomogram model is an effective support tool to predict OS in GC patients, and it can assist researchers and clinicians in determining the appropriate therapeutic strategies for individual patients.

## 4 DISCUSSION

In recent years, the application of radiotherapy has become increasingly common with the development of radiation technology. However, whether PCRT is more beneficial for resectable GC patients than PCT remains unclear, and thus, we aimed to address this in this study. At present, approaches to perioperative therapy differ between Western countries and Asia. In the United States, adjuvant chemotherapy combined with radiotherapy has been recommended as standard care because D2 lymph node dissection is not commonly performed.^27^, ^28^ In Asia, trials are more inclined to include postoperative chemotherapy.^8^, ^29^ Heterogeneity of GC is long considered as an important clinical determinant of patient outcomes. Therefore, based on the clinicopathological features of patients, it is crucial to choose the appropriate treatment strategies in order to improve patient prognosis.^30^ Despite clear evidence showing the benefits of both PCT and PCRT, it is still less clear in which situation PCRT can achieve a better outcome.

Based on a cohort of 6890 cases with locally advanced resectable GC, the univariate Cox regression analyses were performed. Obvious difference between OS and GCSS was seen in the *P*‐value of gender. There are a variety of possible explanations for this finding. First, GCSS measures the proportion of people who are expected to die due to gastric cancer. Unlike overall survival, it excludes death due to causes unrelated to the gastric cancer, which may result in a difference. Nongastric cancer deaths were more common in males, which resulted in the HR of GCSS was lower in males than females. A second possible explanation is that the baseline and clinicopathologic characteristics of both groups are different. The proportion of elderly patients in females was higher than that in males. We included gender into multivariate Cox proportional hazards modeling to minimize biases. The results showed that gender was not an independent prognostic factor for OS and GCSS of GC.

We observed the specific clinicopathological features of patients who can benefit from PCRT. These characteristics include stage III, grade III/IV, diffuse type, DGC, tumor size >34 mm, and lymph node‐positive. For individuals with GC, the above characteristics were thought to be important negative prognostic factors leading to poor survival. Among these features, an important factor is stage III. GC patients with stage III disease and one of the other five features (grade III/IV, diffuse type, DGC, tumor size >34 mm, and lymph node‐positive) at the same time are more likely to benefit from PCRT.

The INT 0116 clinical trial is the milestone of postoperative CRT for GC.^10^ In the test group and control group, 75% and 73% of subjects were Caucasians, respectively. Postoperative CRT resulted in a significant improvement in overall and relapse‐free survival compared with surgery alone. It is worth noting that the INT 0116 trial was unable to detect any significant difference in overall or relapse‐free survival according to the extent of the dissection. Aimed at this problem, the ARTIST trial carried out further research.^31^ In D2‐resected GC, postoperative CRT did not appear to significantly improve the OS and disease‐free survival (DFS) compared with adjuvant chemotherapy alone. Subgroup analyses of patients with node‐positive disease or with intestinal‐type GC revealed a significant increase in DFS in the postoperative CRT group. However, both studies have some limitations. The deficiencies of INT 0116 are mainly the lack of uniformity of surgical techniques (only 10% of cases underwent formal D2 dissection) and the backwardness of radiotherapy technology. The ARTIST adjuvant CRT study in the Asian population indicated that up to 60% of the patients was diagnosed as stage I‐II. Adjuvant chemotherapy may be sufficient for them, adjuvant CRT is an excessive medical treatment. These differences may have an impact on the effectiveness of postoperative CRT. Additionally, it is interesting to note that an improved outcome was obtained by CRT in intestinal‐type individuals, which is not consistent with our findings. Although the reasons remain to be explored, the difficulty in locoregional control of the diffuse type of GC may be associated with survival benefit.^32^ In 2019, the American Society of Clinical Oncology Annual Meeting abstract 4001 reported the results of the ARTIST II study. Compared with tegafur/gimeracil/oteracil (S‐1) plus oxaliplatin (SOX), adjuvant radiotherapy combined with SOX did not increase the survival rate of patients with D2‐resected GC. Researchers considered that the low completion rate of postoperative radiotherapy was an important factor that caused negative results.

Based on the above clinical trials, it is likely that the negative results of the trials from the East may be explained by the wide use of D2 dissection. Sasako et al found that adjuvant chemotherapy alone could not significantly increase the survival for patients with IIIB GC after D2 dissection, suggesting that there remained some room for improvement.^33^ Therefore, adding radiotherapy for patients with high‐stage GC, especially stage III, after D2 lymphadenectomy may be necessary. In addition, three ongoing clinical trials, TOPGEAR, Neo‐CRAG, and CRITICS II, are focusing on PCT or a combination with preoperative radiotherapy in populations that can benefit from it.^34^, ^35^, ^36^ A published SEER‐based retrospective study of 21,472 stage I‐IV patients treated between 1988 and 2008 found that patients at advanced stages benefited most from adjuvant radiotherapy with chemotherapy.^37^ Our findings are consistent with this retrospective study. The latest National Comprehensive Cancer Network® (NCCN) guidelines (2019.V4) still recommend that postoperative CRT is acceptable for patients receiving a resection less than D2, and postoperative chemotherapy is suitable for patients with D2 lymphadenectomy. In our view, clinicopathological features determine whether patients require radiotherapy after D2 dissection. For patients with an advanced stage or other high‐risk clinical characteristics, such as positive lymph nodes or grade III/IV, radiotherapy can be considered as an important option during the perioperative period.

In routine clinical practice, TNM staging system is the main method of prognostic evaluation for patients with malignant tumors. However, the current staging system is inadequate for prognosis because patients with the same cancer stage have different clinical outcomes.^38^, ^39^ Thus, an accurate clinical prognostic tool specially designed for GC is essential. In this study, we built and assessed a nomogram model for individually predicting survival benefits. The nomogram incorporated demographics, clinical characteristics, and treatment information, which was based on six variables: age at diagnosis, grade, tumor size, the 8th T stage, the 8th N stage and comprehensive treatment. This nomogram revealed good discrimination and calibration performance and exhibited more accurate predictive ability than the traditional 8th TNM staging system. Additionally, the new model exhibited more optimal clinical usefulness as assessed by DCA, NRI, IDI, and time‐dependent ROC curve. To our knowledge, we built the first nomogram and online version derived from a large population‐based database for predicting survival in patients with resectable stage IB‐IIIC GC receiving radiotherapy and chemotherapy. The new model is accurate, reliable, and easy‐to‐use. In summary, this large population‐based work can predict the survival benefits of patients with resectable GC who undergo radiotherapy and/or chemotherapy, and as a result, it may have important clinical influence.

Although this study has many advantages, there remain some limitations. First, as a retrospective study, the inherent selection bias is inevitable. Then, pathological stage is influenced by neoadjuvant treatment and may not be possible to accurately predict who will benefit from therapy. Third, the SEER database does not provide detailed treatment information, such as the proportion of D2 lymphadenectomy, surgical margins, and chemotherapy regimens. Therefore, a prospective controlled study with complete and detailed information is needed in the future to clarify the clinical significance.

There has been a constant search for new treatment strategies for GC. With the development of science and technology, gastric cancer molecular classification, tumor markers, and circulating tumor cells are included in prognostic factors. Advances in genetic technology have led to more accurate identification of GC subtypes. It is worth considering whether the GC classification can guide future perioperative treatment in the era of genotyping.

In conclusion, this large population‐based study revealed factors associated and not associated with survival in patients with resectable stage IB‐IIIC GC. Our work showed that the application of PCRT should be determined according to the clinicopathological features of patients. We further constructed and validated a reliable and practical survival‐predicting nomogram model to accurately predict individualized survival probability and screen patients who were suitable for PCRT. The nomogram, with its user‐friendly online web server, is a novel and precise individualized survival estimation tool for GC patients.

## CONFLICT OF INTEREST

The authors have no conflict of interest.

## AUTHORS CONTRIBUTIONS

JW conceived and designed the study. KYC, FCL, and NDW collected clinical data and performed the statistical analysis. KYC and QL performed the research and wrote the paper. JW and LY reviewed and edited the manuscript. All authors read and approved the manuscript.

## ETHICAL STATEMENTS

The study used de‐identified data and adhered to the World Medical Association's Declaration of Helsinki for Ethical Human Research.

## Supporting information

Fig. S1

Fig. S2

Fig. S3

Table S1
Table S2

## Data Availability

The data that support the findings of this study are openly available in the Surveillance Epidemiology and End Results Program at https://seer.cancer.gov/.
