# Supplementary figures and images for: Individualized prediction of survival benefits from perioperative chemoradiotherapy for patients with resectable gastric cancer
Source: Cancer Med. 2020 Aug 18;9(19):7137–50. doi: 10.1002/cam4.3350 (PMC7541150; doi:10.1002/cam4.3350)

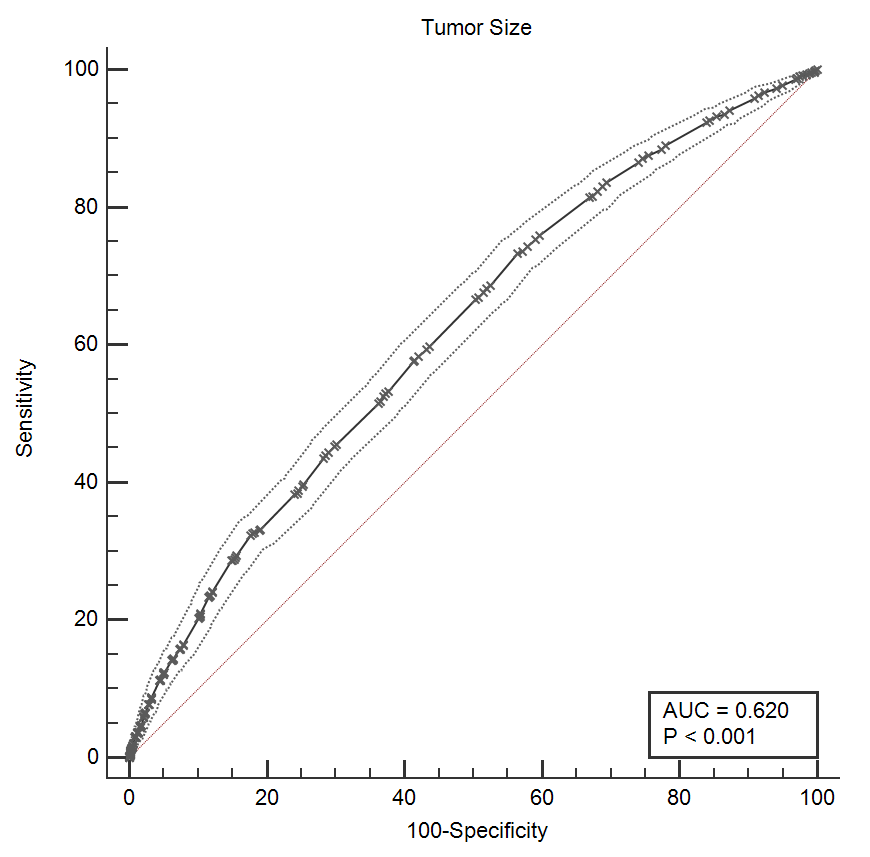

Supplement: Supplementary file 1 — Fig. S1 [file CAM4-9-7137-s001.tif]

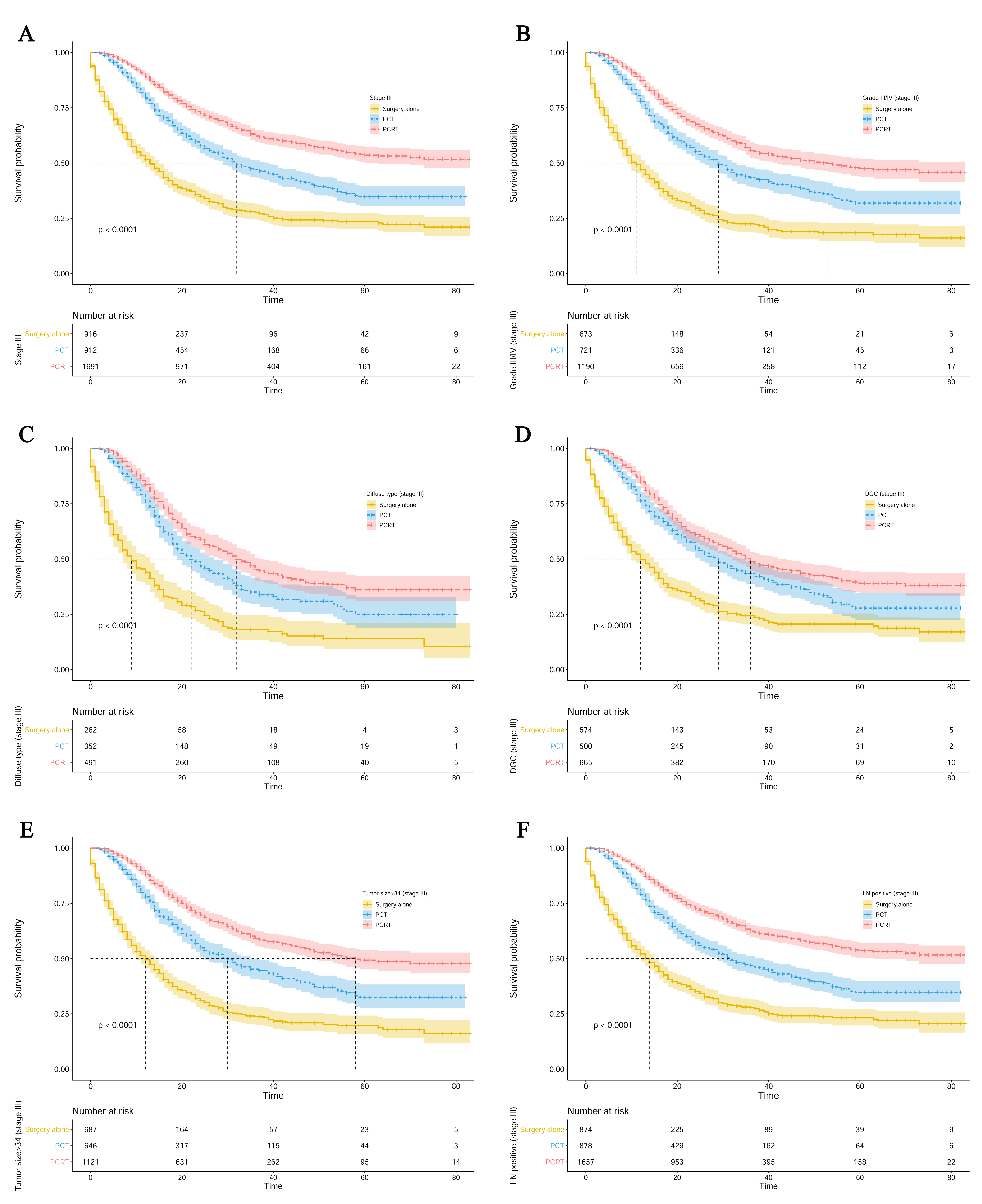

Supplement: Supplementary file 2 — Fig. S2 [file CAM4-9-7137-s002.tif]

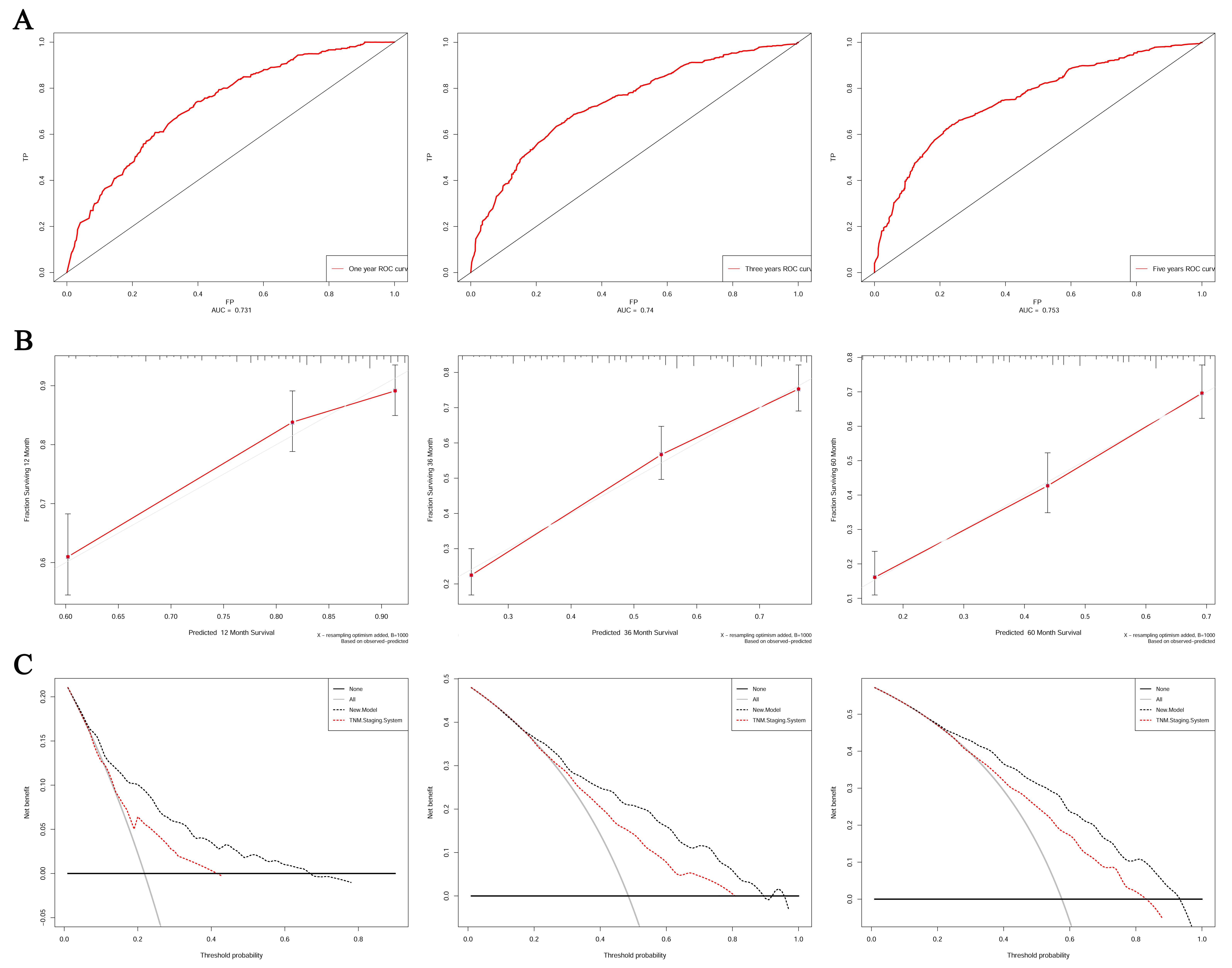

Supplement: Supplementary file 3 — Fig. S3 [file CAM4-9-7137-s003.tif]
