# Supplementary material for: Individualized prediction of survival benefits from perioperative chemoradiotherapy for patients with resectable gastric cancer
Source: Cancer Med. 2020 Aug 18;9(19):7137–50. doi: 10.1002/cam4.3350 (PMC7541150; doi:10.1002/cam4.3350)
Supplement: Supplementary file 4 — Table S1 Table S2 [file CAM4-9-7137-s004.doc]

**Table S1. Histologic Subtypes of Microscopically Confirmed, Gastric Cancer, 2010-2015**

| **Histologic type** | **ICD-O-3 codes** | **Count (%)** |
| --- | --- | --- |
| **Adenomas and adenocarcinomas** | **8140-8389** | **10,260 (76.6)** |
| Adenocarcinoma, NOS | 8140 | 6,692 |
| Linitis plastica | 8142 | 95 |
| Adenocarcinoma, intestinal type | 8144 | 1,601 |
| Carcinoma, diffuse type | 8145 | 809 |
| Adenocarcinoma in adenomatous polyp | 8210 | 36 |
| Tubular adenocarcinoma | 8211 | 99 |
| Carcinoid tumor, NOS | 8240 | 288 |
| Neuroendocrine carcinoma, NOS | 8246 | 166 |
| Adenocarcinoma with mixed subtypes | 8255 | 377 |
| Papillary adenocarcinoma, NOS | 8260 | 23 |
| Other |  | 74 |
| **Squamous cell neoplasms** | **8050-8089** | **136 (1.0)** |
| Squamous cell carcinoma, NOS | 8070 | 115 |
| Squamous cell carcinoma, keratinizing, NOS | 8071 | 11 |
| Squamous cell carcinoma, large cell, nonkeratinzing | 8072 | 3 |
| Squamous cell carcinoma, spindle cell | 8074 | 1 |
| Other |  | 6 |
| **Cystic, mucinous and serous neoplasms** | **8440-8499** | **2715 (20.3)** |
| Mucinous adenocarcinoma | 8480 | 263 |
| Mucin-producing adenocarcinoma | 8481 | 51 |
| Signet ring cell carcinoma | 8490 | 2,401 |
| **All others** | **8010-8049, 8500-8549, 8560-8579** | **290 (2.2)** |
| Carcinoma, NOS | 8010 | 116 |
| Large cell carcinoma | 8012-8014 | 23 |
| Carcinoma, undifferentiated, NOS | 8020 | 18 |
| Small cell carcinoma, NOS | 8041 | 15 |
| Adenosquamous carcinoma | 8560 | 58 |
| Other |  | 60 |
| **Total** |  | 13,401 |

Abbreviations: NOS, not otherwise specified.

**Table S2. Comparison of** **C-indexes Between the Nomogram and 8^th^ TNM Staging System in Gastric Cancer Patients**

| **Stage Types** | **Development Dataset** | | | **Validation Dataset** | | |
| --- | --- | --- | --- | --- | --- | --- |
|  | **C-index** | **95% CI** | ***p* Value** | **C-index** | **95% CI** | ***p* Value** |
| **Nomogram** | 0.702 | 0.693-0.710 | 0.009 | 0.712 | 0.694-0.730 | 0.018 |
| **8th TNM Staging System** | 0.648 | 0.638-0.657 | 0.009 | 0.671 | 0.653-0.690 | 0.018 |

Abbreviations: CI, confidence interval; M, metastasis; N, node; T, tumor.
